# The HMI™ module: a new tool to study the Host-Microbiota Interaction in the human gastrointestinal tract *in vitro*

**DOI:** 10.1186/1471-2180-14-133

**Published:** 2014-05-22

**Authors:** Massimo Marzorati, Barbara Vanhoecke, Tine De Ryck, Mehdi Sadaghian Sadabad, Iris Pinheiro, Sam Possemiers, Pieter Van den Abbeele, Lara Derycke, Marc Bracke, Jan Pieters, Tom Hennebel, Hermie J Harmsen, Willy Verstraete, Tom Van de Wiele

**Affiliations:** 1 Laboratory of Microbial Ecology and Technology (LabMET), Ghent University, Coupure Links 653, B-9000 Gent, Belgium; 2 Laboratory of Experimental Cancer Research, Ghent University, 1P7, De Pintelaan 185, B-9000 Gent, Belgium; 3 Department of Medical Microbiology, University Medical Center Groningen, University of Groningen, Groningen, The Netherlands; 4 ProDigest, Technologiepark 3, 9052 Gent, Belgium; 5 Upper Airways Research Laboratory, Ghent University, Medical Research Building O, De Pintelaan 185, B-9000 Gent, Belgium; 6 Department of Biosystems Engineering, Ghent University, Coupure Links 653, B-9000 Gent, Belgium

**Keywords:** Bacterial adhesion, Enterocytes, Shear stress, SHIME®

## Abstract

**Background:**

Recent scientific developments have shed more light on the importance of the host-microbe interaction, particularly in the gut. However, the mechanistic study of the host-microbe interplay is complicated by the intrinsic limitations in reaching the different areas of the gastrointestinal tract (GIT) *in vivo*. In this paper, we present the technical validation of a new device - the Host-Microbiota Interaction (HMI) module - and the evidence that it can be used in combination with a gut dynamic simulator to evaluate the effect of a specific treatment at the level of the luminal microbial community and of the host surface colonization and signaling.

**Results:**

The HMI module recreates conditions that are physiologically relevant for the GIT: i) a mucosal area to which bacteria can adhere under relevant shear stress (3 dynes cm^−2^); ii) the bilateral transport of low molecular weight metabolites (4 to 150 kDa) with permeation coefficients ranging from 2.4 × 10^−6^ to 7.1 × 10^−9^ cm sec^−1^; and iii) microaerophilic conditions at the bottom of the growing biofilm (PmO_2_ = 2.5 × 10^−4^ cm sec^−1^). In a long-term study, the host’s cells in the HMI module were still viable after a 48-hour exposure to a complex microbial community. The dominant mucus-associated microbiota differed from the luminal one and its composition was influenced by the treatment with a dried product derived from yeast fermentation. The latter - with known anti-inflammatory properties - induced a decrease of pro-inflammatory IL-8 production between 24 and 48 h.

**Conclusions:**

The study of the *in vivo* functionality of adhering bacterial communities in the human GIT and of the localized effect on the host is frequently hindered by the complexity of reaching particular areas of the GIT. The HMI module offers the possibility of co-culturing a gut representative microbial community with enterocyte-like cells up to 48 h and may therefore contribute to the mechanistic understanding of host-microbiome interactions.

## Background

The microbial community inhabiting the human gastrointestinal tract (GIT) can be seen as an additional organ within the body able to produce key factors and bring about specific metabolic pathways within the human body [1-3]. Overall, the structure and composition of this ecosystem reflects a natural selection at both microbial and host levels in order to develop cooperation aimed at functional stability [4]. This interaction mainly occurs at the interface of the mucus and epithelial cell barrier and may influence the regulation of host’s immune and hormonal systems [5-8].

This close cross-talk is a complex area of study due to the limited accessibility of the human GIT and the intrinsic limitations in recreating *in vitro* conditions relevant for an *in vivo*-like interaction [9,10]. In the last two decades, the need for systems that closely mimic the *in vivo* situation led to the creation of dynamic *in vitro* simulators in an attempt to reproduce the physiological parameters of the GIT environment that influence the GI microbial community and its metabolic activity [11-13]. Both the European Food Safety Authority (EFSA) and the US Food and Drug Administration (FDA) support, as a complementary tool, the use of the *in vitro* approach in order to provide evidence of the mechanisms by which a food/constituent could exert the claimed effect, and of the biological plausibility of the specific claim (as reported in the respective guidance). The most intensively used gut simulators include the three-stage continuous culture system, the SHIME® (Simulator of the Human Intestinal Microbial Ecosystem), the EnteroMix, the Lacroix model and the TIM-2 device [14]. Although these systems offer a good reproducibility in terms of analysis of the luminal microbial community [10,14,15], other aspects, such as adhesion of bacteria and host-microbiota interaction are not systematically addressed [16]. Adhesion can be evaluated by means of cell immobilization in anaerobic continuous-flow cultures [17,18]; by encasing mucin beads within a dialysis membrane [19]; by introducing sterile porcine mucin gels in small glass tubes [20] or on plastic carriers (M-SHIME) [21] to determine how intestinal bacteria colonize and degrade mucus. The evaluation of the host’s response is normally conducted by means of cell culture experiments in plates [22], in a trans-well setup [23], by the three-dimensional organotypic model of human colonic epithelium [24] or using the so-called human gut-on-a-chip [25]. Independently of the used approach, the combination of cell lines with complex microbial communities (i.e. gut microbiota) is limited by the fact that bacteria are highly cytotoxic for the cells, thus limiting the experimental time to a few hours [10]. Finally, none of the available devices offers the opportunity of studying the gut biofilm formation and, at the same time, the host-microbiota interaction under continuous simulated conditions.

To overcome these limitations, we propose the use of the Host Microbiota Interaction (HMI) module, taking into account the particular characteristics of the host-microbiota interface in the GIT. More specifically, the aim was to establish a model that allows long-term studies of a complex microbial community colonizing a mucus layer, while being co-cultured - up to 48 h - microaerophilically in the presence of shear forces and a monolayer of enterocyte human cells. We first characterized a number of technical parameters of the HMI module, and then we used the novel device together with the SHIME® to evaluate the possibility of using the HMI module for long-term studies of host-bacteria interactions. The SHIME® consists of a succession of five reactors simulating both the upper and the lower digestive tract, with the first two reactors, mimicking the stomach and small intestine, and the last three compartments simulating physiological and microbiological parameters representative of ascending, transverse and distal colon. We used, as a test compound, a dried product derived from *Saccharomyces cerevisiae’s* fermentation that has already been shown to have immune modulating/anti-inflammatory properties both *in vitro* and in human clinical trials [26-29]. We followed the effect of the treatment on the composition of the luminal and mucosa-associated microbial community and on the simulated host’s response in terms of interleukin-8 production (a pro-inflammatory cytokine produced by enterocytes in response to bacterial triggers).

## Results and discussion

The gut microbiome is an additional organ within our body. To manage this complex community involved in key functionalities for human health, it is important to understand how bacteria interact with the host. This is not always easy due to limited *in vivo* accessibility of the GIT, particularly of the mucosal environment. In this study, we introduced a new methodology to study the host-microbe interaction under controlled *in vitro* conditions.

### The HMI module

**Figure 1:**
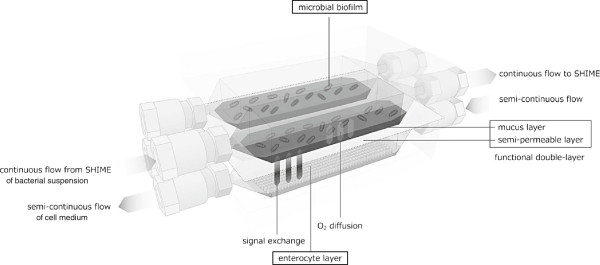
**Scheme of the HMI module for long-term studies of the host-microbiota interaction in the GIT.** A polyamide semipermeable membrane and a mucus layer form a double functional layer that separates the luminal compartment (upper one) from the lower compartment containing enterocyte cell lines. The HMI module allows to study the bacterial adhesion under relevant shear forces and microaerophilic conditions. It allows the reciprocal exchange of signals and metabolites between compartments and it allows the exposure of cell lines to a complex microbial community, representative for the human colon, for up to 48 h.

A new *in vitro* model, i.e. HMI module, was developed to study the indirect host-microbe interaction in the gastrointestinal tract. It comprises two parallel setups in order to perform experiments in duplicate, with each setup consisting of two compartments separated by a functional double-layer (Figure 1). The upper compartment simulates the luminal side of the GIT (with a mixed microbiota from the SHIME), whereas the lower compartment contains enterocytes, simulating the host. Further, the functional double layer is composed of an upper mucus layer and a lower semi-permeable polyamide membrane and has been conceived to potentially serve multiple objectives: i) to provide a mucosal area which can be colonized by the gut bacteria; ii) to allow the bilateral transport of low molecular weight metabolites; iii) to allow the transport of oxygen from the lower to the upper side of the mucosal layer in order to create microaerophilic conditions at the bottom of the growing biofilm; and iv) to protect the host’s cells from direct exposure to a complex microbial community and its toxic effects. In this study the HMI module has been used in i) short-term experiments to characterize different technical parameters and ii) in a long-term experiment, coupled to a SHIME system (as described in the related paragraph), to assess the possibility to follow up the host’s response to a specific treatment up to 48 h.

### Characterization of the technical parameters (shear stress, mucus thickness and oxygen diffusion)

In the first part of the work the newly developed model has been characterized with respect to a number of technological parameters in order to validate it with *in vivo* data. For these experiments the HMI module has been used as a separate unit (i.e. not coupled with a SHIME).

The optimal shape of the HMI module was designed to provide a homogeneous fluid shear distribution on the surface of the mucus layer under different shear forces relevant for the GIT (Additional file 1: Figure S1). Analysis by Confocal Laser Scanning Microscopy (CLSM) of the mucus layer on a vertical section and the evaluation of the mucus thickness showed that 95% (i.e. residual thickness) of the original mucus layer (200 μm) was still present after 5 hours at medium shear stress (10 dynes/cm^2^) and 45% after high shear stress (20 dynes/cm^2^) (data not shown). Shear forces in the gut are a key factor in shaping the adhering community, in affecting bacterial gene expression and physiology, and can alternatively favor or disfavor the adhesion of specific strains [30-32]. Physiological levels of shear stress found in the intestinal epithelium during peristalsis may range between 35 and 0.02 dynes/cm^2^[25,33,34]. We chose values below 5 dynes/cm^2^ as these are representative for the final part of the ileum and beginning of the proximal colon. In fact, in absence of microvilli, the fluid shear stress would vary from about 1 to 5 dynes/cm^2^[35].

Once the shape of the model and the flow were established, we assessed the capacity of metabolites and oxygen to permeate through the double functional layer of the HMI module. A water solution containing FITC dextran was flown in the upper compartment and samples were collected from the lower compartment to measure the fraction of fluorescent product that could permeate through the double functional layer. The experiment was conducted without and with a 200 μm mucus layer on the membrane. The permeability coefficients ranged from 2.4 × 10^−6^ cm sec^−1^ for the 4 kDa dextran to 7.1 × 10^−9^ cm sec^−1^ for the 150 kDa dextran (Table 1), demonstrating an inverse relationship between the size of the metabolite and the degree of permeation. When comparing modules with and without mucus layer, the presence of mucus further induced a decrease in the permeability of the test product (Table 1), as also shown by Desai et al. [36]. The obtained values are in the same range of other studies conducted with Caco-2 cells [25], perfused animals [37] or *ex-vivo* human colon tissues [38]. Behrens et al. [39] reported that undifferentiated HT-29 cells have a high permeability for 4 kDa dextrin (7 × 10^−6^ cm sec^−1^) which decreases with increasing thickness of mucus to 1 × 10^−6^ cm sec^−1^. A similar setup was used to assess the oxygen permeation through the double functional layer (mucus thickness of 200 μm). In this case O_2_-saturated water (8.5 mg/L) was added in the lower compartment while deoxygenized water was added in the upper compartment. The oxygen concentration was then measured in the upper compartment: an oxygen permeability (PmO_2_) of 2.5 × 10^−4^ cm sec^−1^ resulted in a diffusion coefficient (DO_2_) of 5.0 × 10^−6^ cm^2^ sec^−1^. The PmO_2_ value obtained with the HMI module was in line with the *ex vivo* theoretical permeability diffusion calculated by Saldena and colleagues [40] for a mucus layer of 115 μm (i.e. PmO_2_ = 2.1 ⋅ 10^−4^ cm sec^−1^).

**Table 1:** **Permeability coefficients for metabolites and oxygen (PmO**_**2**_**) in presence of a polyamide membrane (pore size 0.2 μm) with and without mucus layer (200 μm) (n = 2)**

**Polyamide membrane**	***FITC dextran***			***Oxygen***
	***4 kDa***	***20 kDa***	***150 kDa***	
With mucus	2.4 ± 10^−6^	2.5 ± 10^−7^	7.1 ± 10^−9^	2.5 ± 10^−4^
Without mucus	5.6 ± 10^−6^	4.1 ± 10^−7^	6.5 ± 10^−7^	ND^a^

^a^ND = not determined.

Data are expressed as cm sec^−1^. The permeation coefficient was lower in presence of the mucus and with the increase of the FITC dextran kDa.

### Characterization of the biological parameters

A final set of short-term experiments was conducted to assess the capability of bacteria to colonize the mucus layer (200 μm) and to evaluate the survival of the enterocytes in the lower compartment when exposed to a complex microbiota.

In the first setup, a pure culture of *Lactobacillus rhamnosus* GG (LGG) was flown in the upper compartment. LGG was chosen as a positive control, because human *in vivo* studies showed that the beneficial effects of LGG are, in part, attributed to a strong colonization of the colonic mucus layer upon oral administration [41]. This strong adhesion capacity of LGG has recently been attributed to a SpaC pilin, which is located on the top of the pili and exerts a strong mucus-binding activity [42]. After 1.5 h of incubation in the upper compartment of the HMI module, LGG showed an adhesion percentage of 15.7 ± 3.2%, as compared to the original concentration dosed to the model. This value is in line with what described by Van den Abbeele et al. [21], who tested the adhesive properties of LGG in presence of a complex gut microbiota in a M-SHIME. The colonization capacity of mucus by LGG was thus confirmed in the HMI module.

**Figure 2:**
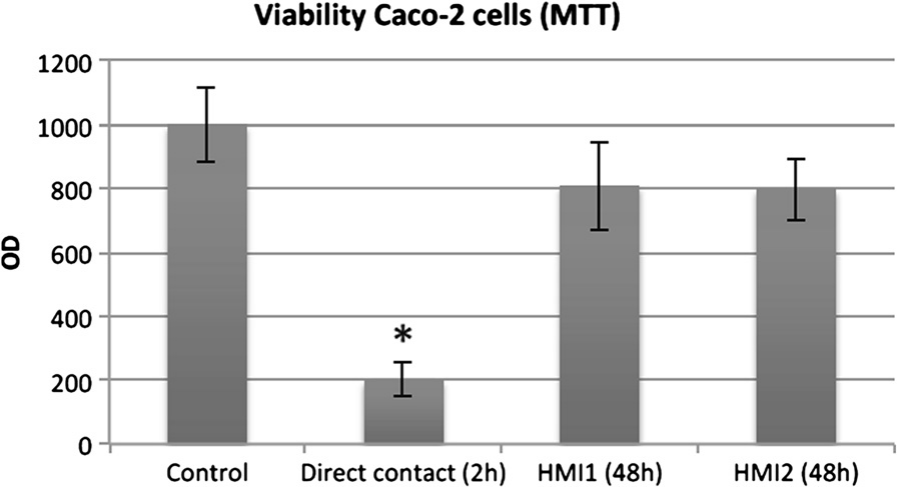
**MTT values (expressed as Optical Density – OD) of Caco-2 cells directly exposed for 2 h to the complex microbial community of the ascending colon of a SHIME reactor (direct contact), exposed to the same microbial community within a HMI module (HMI 1 and 2) or to sterile SHIME medium (control) for 48 h.** Values are averages ± standard deviation (n = 2). * = statistically different from the control condition according to a Student’s two-tailed t-test (p < 0.05).

Finally, the HMI module containing enterocytes in the lower compartment was challenged for the first time with a complex microbiota originated from the simulated ascending colon of the SHIME. In parallel the enterocytes were also directly exposed to the same complex microbiota. A MTT test showed that the viability of Caco-2 cells directly exposed to the complex microbial community decreased by 80% after 2 hours of co-culture. In contrast, when the interaction occurred within an HMI module, the cells’ viability after 48 h of incubation was not significantly different as compared to a control system in which only sterile SHIME medium was dosed (Figure 2). Although the use of cell cultures, such as Caco-2 cells, is not novel for mechanistic studies [29,43,44], the output of these reductionist studies is limited by the fact that they are normally conducted using pure bacterial cultures, a mix of few bacterial strains or filtered growth media. This is mainly related to the fact that mixed microbial slurries are too cytotoxic (Figure 2), thus limiting the experimental time (a few hours at most) and the adaptation of the host to the microbial metabolism. On the contrary, the HMI module allows to indirectly expose the Caco-2 cells to the gut microbiota for up to 48 h, the average *in vivo* exposure time of an enterocyte to the content of the gut lumen when migrating from the crypts to the top of the villi [45].

### Long-term experiment

**Figure 3:**
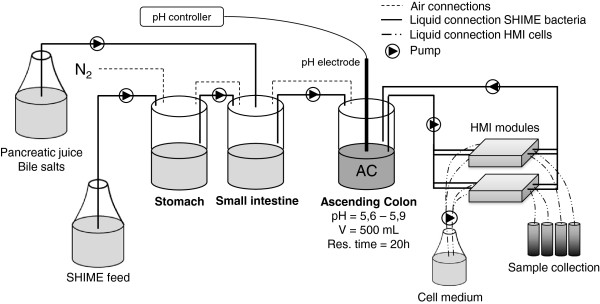
**Scheme of the adapted SHIME system (consisting of stomach, small intestine and ascending colon - AC - compartments) used for the long-term study.** Two HMI modules have been connected in parallel to the vessel simulating the AC compartment in order to obtain information on bacterial adhesion and host response after 24 and 48 h. The SHIME system was fed three times per day with SHIME feed; the medium in the lower compartment of the HMI modules (containing Caco-2 cells) was fully replaced every 6 hours by means of an automatic pump. The exhausted medium was collected in order to analyze the concentration of IL-8.

**Figure 4:**
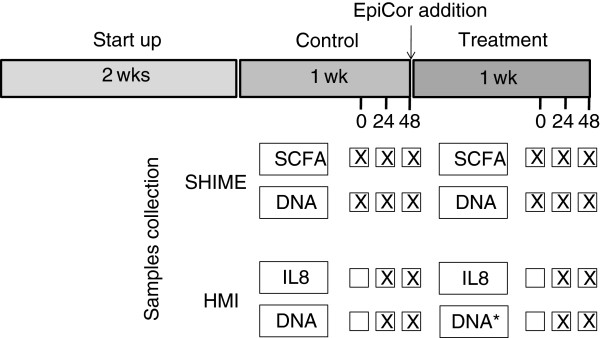
**Scheme of the long-term experiment and of the relative sampling points for the different analyses.** The experiment consisted of a 2-week startup period, 1-week control and 1-week treatment. The HMI modules were connected to the ascending colon compartment of a SHIME system during the last 3 days of the control and treatment periods. Samples from the lumen of the SHIME were collected for SCFA and DNA analyses. Samples from the surface of the double functional layer of the HMI modules were collected for DNA analyses. Samples from the lower compartment of the HMI module were collected for IL-8 measurements. DNA = qPCR and DGGE. DNA* = qPCR, DGGE and FISH (the latter only at 48 h).

After the validation of the technical/biological parameters in independent experiments, the HMI module was used in combination with an adapted SHIME (Figure 3) to test the effect of a dried, modified *Saccharomyces cerevisiae* fermentation product (EpiCor®, Embria Health Sciences, USA). The adapted SHIME consisted of a succession of three reactors: the first two reactors are of the fill-and-draw principle to simulate different steps in food uptake and digestion by simulating, respectively, stomach and small intestine; the last compartment, simulating the ascending colon (AC), was a continuously stirred reactor with constant volume, pH control and inoculation with fecal microbiota. As described in more detail in the ‘Methods’ section, two HMI modules were connected to the AC vessel of the SHIME during the last three days of the control and of the treatment week (Figures 3 and 4).

Considering the average of three sampling points in the SHIME experiment (Figure 4), the treatment with the dried-fermented yeast product induced a 35% increase in total short chain fatty acids (SCFA) production in the lumen of the simulated AC (from 73.6 ± 1.4 to 99.7 ± 3.5 mmol/L) with a 41% increase of acetate (from 37.8 ± 2.4 to 53.2 ± 2.4 mmol/L), a 6% increase of propionate (from 17.0 ± 1.0 to 18.1 ± 1.1 mmol/L) and a 31% increase of butyrate (from 13.6 ± 0.5 to 17.8 ± 0.6 mmol/L) (p < 0.05).

Quantitative PCR data at luminal level in the AC showed that at the moment of connecting the HMI module to the SHIME during the treatment period, the concentration of all the analysed microbial groups was lower as compared to the respective time point during the control period. Despite this, at the end of the 48 h-treatment period, the bacteria concentration of all groups were equal or higher than the respective sampling points during the control period (Table 2). At mucosal level, all microbial groups at the end of the treatment showed a lower concentration with the exception of the *Lactobacillus* spp. which, at 48 h, was 2 log higher as compared to the same time point during the control period.

**Table 2:** Bacterial concentration of different microbial groups quantified by specific qPCR in luminal (L) (n = 3) and mucosal (M) (n = 6) samples of the HMI module during control and treatment at time 0, 24 and 48 h

		**Control (A)**					**Treatment (B)**				
		**0 h**	**24 h**		**48 h**		**0 h**	**24 h**		**48 h**	
		**L**	**L**	**M**	**L**	**M**	**L**	**L**	**M**	**L**	**M**
**Total Bacteria**	Avg.	2.46 × 10^10^	1.31 × 10^10^	5.71 × 10^8^	9.08 × 10^9^	6.35 × 10^8^	*6.35 × 10*^*9*^	*6.27 × 10*^*9*^	*2.43 × 10*^*8*^	7.79 × 10^9^	*2.31 × 10*^*7*^
	Std. Dev.	1.12 × 10^9^	1.53 × 10^8^	2.83 × 10^8^	4.77 × 10^8^	8.44 × 10^8^	3.14 × 10^8^	7.54 × 10^7^	1.75 × 10^8^	2.29 × 10^8^	2.56 × 10^6^
**Bacteroidetes**	Avg.	7.60 × 10^9^	6.29 × 10^9^	5.25 × 10^8^	4.58 × 10^9^	2.78 × 10^8^	*1.41 × 10*^*9*^	4.50 × 10^9^	*9.82 × 10*^*7*^	**1.13 × 10**^**10**^	6.59 × 10^7^
	Std. Dev.	1.23 × 10^9^	2.77 × 10^9^	3.60 × 10^8^	1.20 × 10^9^	3.65 × 10^8^	1.83 × 10^8^	6.96 × 10^8^	6.07 × 10^7^	1.79 × 10^9^	3.44 × 10^7^
**Firmicutes**	Avg.	1.65 × 10^9^	1.64 × 10^8^	2.08 × 10^7^	2.85 × 10^8^	1.67 × 10^7^	*7.88 × 10*^*8*^	**4.29 × 10**^**8**^	3.65 × 10^6^	**5.43 × 10**^**8**^	*9.65 × 10*^*5*^
	Std. Dev.	2.79 × 10^8^	1.02 × 10^7^	3.80 × 10^6^	2.52 × 10^7^	3.20 × 10^6^	7.21 × 10^7^	3.96 × 10^7^	1.60 × 10^6^	4.11 × 10^7^	7.41 × 10^5^
**Bifidobacteria**	Avg.	9.39 × 10^8^	2.73 × 10^8^	3.35 × 10^8^	3.24 × 10^8^	8.49 × 10^6^	*1.26 × 10*^*8*^	3.79 × 10^8^	*1.25 × 10*^*6*^	**4.43 × 10**^**8**^	*3.37 × 10*^*5*^
	Std. Dev.	1.23 × 10^8^	2.65 × 10^7^	5.09 × 10^7^	2.97 × 10^7^	9.80 × 10^5^	2.89 × 10^7^	1.40 × 10^8^	1.38 × 10^5^	2.44 × 10^7^	1.74 × 10^5^
**Lactobacilli**	Avg.	1.88 × 10^7^	3.86 × 10^6^	1.30 × 10^5^	6.81 × 10^5^	3.45 × 10^2^	*8.06 × 10*^*5*^	*1.77 × 10*^*5*^	*1.45 × 10*^*3*^	1.37 × 10^6^	**5.85 × 10**^**4**^
	Std. Dev.	3.47 × 10^6^	3.45 × 10^5^	7.75 × 10^4^	5.40 × 10^5^	3.89 × 10^2^	1.69 × 10^5^	1.54 × 10^5^	1.67 × 10^3^	2.52 × 10^5^	7.86 × 10^4^

Data for L are expressed as 16S rRNA gene copies/mL of SHIME suspension; those for M correspond to 16S rRNA gene copies cm^−2^ of simulated gut wall. Values in bold indicate samples from the treatment period which are significantly higher than the control at the same sampling time, according to a Student’s two-tailed t test (p < 0.05). Values in italics are significantly lower.

**Figure 5:**
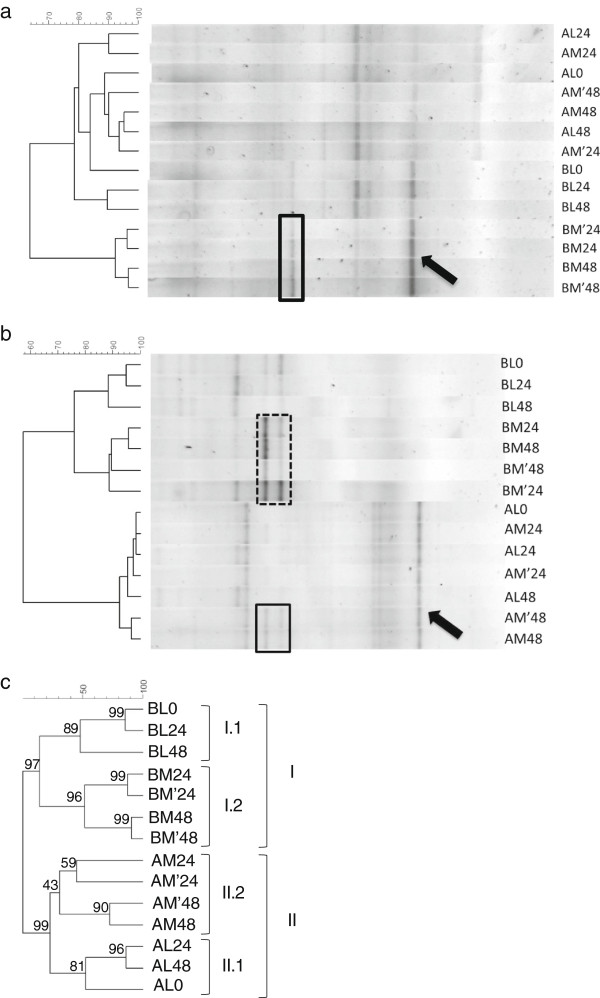
**DGGE fingerprinting analysis for bifidobacteria (a) lactobacilli (b) and composite data set of the gels for bifidobacteria.** lactobacilli and total bacteria, including bootstrap analysis with 1000 samplings **(c)**. A = control period (Cluster II); B = treatment period (Cluster I). L = luminal samples collected from the SHIME reactor; M = mucus sample collected from a fraction of the membrane inside the HMI module. 0, 24 and 48 indicate the hours that the HMI modules have been connected to the SHIME system during the control and treatment periods (as illustrated in Figure 3). Clustering analysis was based on the Pearson product–moment correlation coefficient and dendrograms were created by using UPGMA linkage.

The cluster analysis based on a composite data set of the DGGE gels for total bacteria (Additional file 1: Figure S2), bifidobacteria (Figure 5a) and lactobacilli (Figure 5b) is shown in Figure 5c. The samples from control and treatment period clustered separately (cluster I and II). Moreover, within each cluster, luminal samples and mucosal samples sub-clustered in two different groups (Figure 5c). The DGGE specific for bifidobacteria (Figure 5a) showed that two distinct *Bifidobacterium spp.* – indicated by an arrow and a black square could benefit from the treatment and specifically colonize the mucus layer. The *Bifidobacterium* sp. identified by the black square was only dominant in the microbial biofilm during the week of treatment. Also for lactobacilli, the DGGE showed qualitative changes due to the treatment (Figure 5b). The *Lactobacillus* sp. indicated by the black arrow, initially present both in the luminal and the mucosal microbial community, were lost during the treatment. On the contrary, the treatment selectively enhanced those species within the dashed square, species that preferentially adhere to the simulated gut surface. These molecular data showed that by means of an HMI module connected to the SHIME, it was possible to evaluate the modulating effect of the test product both on the luminal and mucosa-associated microbiota. The latter was different from the luminal one (in terms of relative abundance of the main species) as the mucin layer is colonized by a biofilm with bacterial species that specifically (i) adhere to mucins, (ii) metabolize mucins or (iii) proliferate in mucus due to the microaerophilic conditions at the bottom of this layer. This is also the case *in vivo*, where it was shown for instance that the mucosa-associated microbiota differs from the dominant fecal microbiota in both healthy subjects and patients with IBD [46].

**Figure 6:**
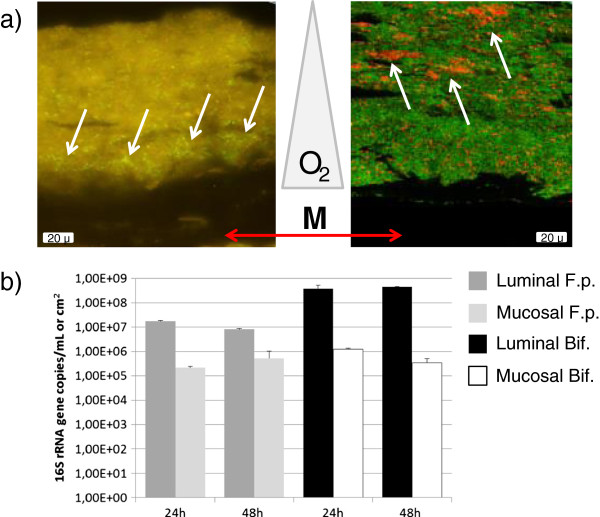
**FISH analyses a) positioning of *F. prausnitzii *(left panel - fluorescent microscopy) and bifidobacteria (right panel - Confocal Laser Scanning Microscopy) in the microbial biofilm with respect to the membrane and mucus layer (M), as indicated by the white arrows.** Oxygen concentration (O_2_) is assumed to decrease from the bottom to the top of the biofilm. The green background is auto-fluorescence of the matrix: EPS, and non-responding bacteria in the left panel, while in the right panel it corresponds to bacteria stained with the EUB338 probe FITC labeled, and also some auto-fluorescent EPS. **b)** Concentration of *F. prausnitzii* (F.p.) and *Bifidobacterium* spp. (Bif.) in the lumen of the SHIME (L) and mucus layer (M) of the HMI module during the treatment period determined by specific qPCR (n = 3).

Finally, the positioning of two specific microbial groups (i.e. bifidobacteria and *Faecalibacterium prausnitzii*) in the mucus layer as analysed by FISH, provided an additional proof of the validity of the HMI module as compared to the *in vivo* situation (Figure 6). While the strict anaerobic bifidobacteria tended to colonize the upper side of the mucus layer, *F. prausnitzii* mainly occurred in the lower part of the mucus, i.e. at the anoxic/oxic interphase (Figure 6a). Khan et al. demonstrated that *F. prausnitzii* can grow in the oxic-anoxic interphase due to the fact that this microorganism, despite being oxygen sensitive, copes with O_2_ because of a special extracellular electron shuttle of flavins and thiols [47]. Similar to the *in vivo* situation - where small amounts of oxygen permeate from blood vessels towards the gut lumen - in the HMI module, oxygen diffusion from the aerobic lower chamber to the anaerobic upper chamber (Figure 1) probably results in microaerophilic conditions at the base of the biofilm, allowing for *F. prausnitzii* to specifically colonize this niche. The qPCR data showed a decreasing concentration of *F. prausnitzii* in the luminal compartment and an increasing one in the mucus layer along the 48 h experiment (Figure 6b). The concentration of bifidobacteria remained unaffected in the luminal part while tended to decrease in the mucus layer compartment. The FISH data thus demonstrate the potency of the HMI module to preserve the regional colonization of specific gut microorganisms within the mucus layer.

**Figure 7:**
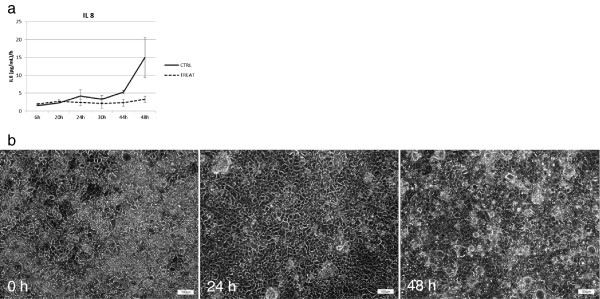
**Cytokine production and enterocytes (a) data related to the IL-8 production along the experiment (n = 2).** Data are expressed as (pg mL^−1^)/h; the standard deviation was calculated on the readings of the two parallel setups. **(b)** Microscopy scans of the cell lines collected from the HMI modules of the SHIME treated with the test product to evaluate the morphology at time 0, 24 and 48 h.

Finally, the possibility of exposing the enterocytes to complex microbial communities for a prolonged period allowed us to follow up the response of the host-like cells to the specific treatment. Figure 7b shows that, after 24 h and 48 h, the morphology of the Caco-2 cells during and at the end of the treatment period was comparable with that of the cells at the beginning of the experiment. Moreover, the cells remained attached as a monolayer to the collagen substrate and were viable (no statistically significant difference in terms of MTT values). The samples collected from the lower chamber when the medium was replaced every 6 h (‘6 h-sample’) were used to assess the residual concentration of O_2_ and the production of IL-8 by Caco-2 cells. The dissolved O_2_ in the fresh cell medium was 8.44 mg L^−1^. This concentration decreased to 7.75 ± 0.06 mg L^−1^ in the ‘6 h-sample’ at 6 h, to 7.25 ± 0.06 mg L^−1^ in the ‘6 h-sample’ at 24 h and to 7.22 ± 0.03 mg L^−1^ in the ‘6 h-sample’ at 48 h. This indicates that the O_2_ concentrations did not decrease dramatically in the lower compartment over time. The treatment with the yeast fermentate resulted in an anti-inflammatory response as evidenced by significant lower IL-8 production after 48 h (p < 0.05), as compared to the control (Figure 7a). The significant decrease in pro-inflammatory IL-8 production has already been correlated with a SCFA profile that shifted towards an increased production of butyrate [29].

## Conclusions

The study of the *in vivo* functionality of adhering bacterial communities in the human GIT and of the localized effect on the host is frequently hindered by the complexity of reaching particular areas of the GIT, and by the lack of suitable *in vitro* models simulating the actual GIT complexity. In order to overcome this limitation we proposed the HMI module as a simplified simulation of the processes occurring at the level of the gut wall (i.e. shear stress, O_2_ and metabolites permeation, bacterial adhesion and host response). Three unique advantages can be ascribed to this new device, as compared to other systems available for research purposes: i) the possibility to simulate at once the bacterial adhesion to the gut wall and the indirect effect on human cell lines; ii) the possibility of performing these studies up to 48 h with a complex microbiota, representative of that inhabiting the human gut; iii) the possibility to couple the HMI module to a continuous simulator of the human gastrointestinal tract (i.e. SHIME). The latter is of key importance when analyzing the effect of specific products, as for instance prebiotic fibers. In fact, the health-modulating effect of fibers is often related to the metabolites produced by microbial species by means of cross-feeding [48,49]. For instance, primary users often degrade part of an ingredient to smaller fragments, sugar monomers, and SCFA such as acetate or lactate. The latter two are precursors for the production of the anti-inflammatory SCFA butyrate by other species [50]. The efficiency of this mechanism is frequently related to the adaptation of the microbial metabolic functionalities to the fiber and, in order to exert this effect, repeated doses of the ingredient are needed [29]. This is exactly what the combination ‘SHIME-HMI module’ allows to study: repeated doses of a product are provided to the microbiota of the SHIME; the product modifies the composition and activity of the luminal and mucosal microbiota and, ultimately, this modulates the host’s response.

Several opportunities lay in the future to improve the host compartment of the HMI module. Among them, the most challenging would be the incorporation of co-cultures of enterocytes and immune cells or of three-dimensional organotypic model of human colonic epithelium [24].

## Methods

### The HMI module

The HMI module consists of 2 compartments (each measuring 10 × 6 cm) separated by a functional double-layer composed of an upper mucus layer and a lower semi-permeable membrane (Figure 1). The upper compartment represents the luminal side of the GIT, whereas the lower compartment contains enterocytes representing the host. The polyamide membrane has a pore size of 0.2 μm and a thickness of 115 μm (Sartorius Stedim, Vilvoorde, Belgium). The mucus layer was prepared by boiling autoclaved distilled H_2_O containing 5% porcine mucin type II (Sigma Aldrich, St. Louis, MO, USA) and 0.8% agar. The pH was adjusted to 6.8 with 10 M NaOH. A mucus layer ranging between 200 and 250 μm (depending on the experiment) [51] was poured onto a wet membrane by means of an Elcometer 4340 Motorised/Automatic Film Applicator (Elcometer SA, Hermalle/s Argenteau, Belgium).

Caco-2 cells (ATCC HTB37) were cultivated in Dulbecco’s Modified Eagle’s Medium (DMEM) supplemented with Glutamax (Gibco), 10% fetal bovine serum (Greiner Bio-One, Wemmel, Belgium), 1% non-essential amino acids and 2% penicillin/streptomycin + 2.8 μg/ml amphotericine B (fungizone) at 5% CO_2_ in air. Before the experiment, 88.000 cells/cm^2^ were seeded on glass slides covered overnight with rat-tail collagen of type I (BD Biosciences, Belgium) and grew till confluence [52]. After 7 days of culture, the cells were ready to be challenged with bacteria. During the experiments, the cells were maintained in the same medium but without antibiotics and antimycotics to avoid killing the bacteria growing in the upper chamber.

### Characterization of the technical parameters

Hydrodynamics studies (computational fluid dynamics) were carried out by means of Fluid 6.0 CFD software (ANSYS, Canonsburg, USA) [53]. The aim was to evaluate the best design for generating a homogeneous flow within the compartments under different shear forces representative of the upper and distal small intestine and of the colon (i.e. 25, 12 and 2 dynes cm^−2^, respectively).

The adhesive capacity of the mucus layer to the polyamide membrane was evaluated by means of CLSM. Two HMI modules were set up and fluorescein isothiocyanate (FITC) dextran (4 KDa), a fluorescent compound, was added to the mucin/agar layer in order to make the mucus visible by CLSM. The integrity of the mucus layer (200 μm) was analyzed after a 5-hour incubation period under either medium or high shear stress (i.e. 10 and 20 dynes/cm^2^). Data were calculated as percentage of residual mucus (after 5 h) on the membrane as compared to Time 0, analyzing a vertical section of the functional double layer.

In a separate experiment, three HMI modules were set up to evaluate the permeation of metabolites of different dimensions and molecular radius through the double functional layer by means of a water solution containing FITC conjugated dextran of 4, 20 and 150 kDa, as model compounds. The permeability of the polyamide membrane was assessed with and without a 200 μm mucus layer and at a constant flow of 6.5 mL min^−1^. A standard curve based on the molar concentration was created for each compound. Measurement of the fluorescent compounds (collected from the lower compartment) at an excitation wavelength of 485 nm and an emission wavelength of 530 nm and calculation of the permeability coefficient (Pc) was conducted as reported in Ambati et al. [54], using the following equation:

$$\mathrm{Pc}=\frac{\left(C3.5-C0.5\right)\times V}{A\times t\times C0}$$

where C3.5 and C0.5 were the concentration of the FITC dextran in the lower compartment at 3.5 h and 0.5 h, respectively, V the volume of the compartment, A the area of the membrane, t the time of the experiment and C0 the initial concentration of FITC dextran in the upper compartment.

The oxygen permeability was measured in a HMI module (with a mucus layer of 200 μm) maintaining a completely anaerobic upper chamber (water previously gassed with 95% N_2_-5% CO_2_) and an aerobic lower chamber (liquid constantly gassed with an air pump). Measurements were carried out at 37°C by following the increasing oxygen concentration in the upper chamber by means of a luminescent LDO oxygen probe (Hach Lange, Mechelen, Belgium) placed on the outlet connection of the luminal side of the module. Data of the increasing oxygen concentration in the upper chamber, collected in the first 30 minutes, were used to calculate the relative permeability (PmO_2_) using the following equation, as shown by Saldena et al. [40]:

$$\mathrm{MO}2=\mathrm{DO}2\times S\times t\times \frac{\left(\mathrm{cO}2\right)A-\left(\mathrm{cO}2\right)B}{x}$$

where MO_2_ is the mass of oxygen transferred in the time *t*; (*c*O_2_)_A_ and (*c*O_2_)_B_ are the concentrations of oxygen in the upper and lower chamber of the HMI module with a mucus layer with a surface S and a thickness *x*. The quotient *D*O_2_/*x* corresponds to the oxygen permeability (*Pm*O_2_).

### Characterization of the biological parameters

*Lactobacillus rhamnosus* GG (LMG 18243, BCCM/LMG, Ghent, Belgium) was used as a positive control to assess the capacity of bacteria to colonize the double functional layer [55]. LGG was grown in MRS medium, quantified by plate count (LGG t0). The fully grown liquid culture was then circulated through the upper chamber of an HMI module at a flow correspondent to a shear stress of 3 dynes cm^−2^ (6.5 mL min^−1^). After 1.5 h, the simulation was stopped and the luminal suspension removed. The functional layer was rinsed twice with phosphate buffer solution to remove the non-adhered bacteria. Subsequently, the luminal side of the functional layer was rinsed with Triton X-100 to remove the adhering bacteria. The obtained bacterial suspension was analyzed for microbial concentration measurements using the plate count technique on MRS (LGG t1.5). Percent of adhering bacteria was calculated as LGG t1.5/LGG t0.

In a second set of experiments, it was evaluated the capacity of Caco-2 cells to survive in the HMI module in presence of a complex microbial community (derived from a SHIME reactor). An HMI module was set up as described in the first paragraph of the *Methods* section and the complex microbial community was introduced in the upper chamber of the HMI module. In a parallel experiment, the enterocytes were directly exposed to the same microbiota (i.e. viability after direct contact) in a microtiter plate. The cell viability in the 2 setups was compared by means of the MTT ((3-(4,5-Dimethylthiazol-2-yl)-2,5-diphenyltetrazolium bromide) colorimetric test [56] after 48 h of incubation in the HMI module and after 2 h of direct contact.

### SHIME experiment

The HMI module was used in combination with an adapted Simulator of the Human Intestinal Microbial Ecosystem (SHIME®) to test the effect of a dried, modified *Saccharomyces cerevisiae* fermentation product (EpiCor®, Embria Health Sciences, Ankeny, Iowa, USA). *S. cerevisiae* is anaerobically fermented in a proprietary medium and the whole medium is dried to inactivate the yeast and then ground to a suitable particle size leading to the following composition for 100 gram of product: carbohydrates 39%, total dietary fiber 11.9%, protein 27.9%, total fat 2.07%, and cholesterol 0.02%. The adapted SHIME consisted of a succession of three reactors [57,58] (Figure 3). The first two reactors are of the fill-and-draw principle to simulate different steps in food uptake and digestion, with peristaltic pumps adding a defined amount of a carbohydrate-based nutritional medium (140 mL 3 time/day) and pancreatic and bile liquid (60 mL 3 times/day), respectively to the stomach and duodenum compartment and emptying the respective reactors after specified intervals. The last compartment is a continuously stirred reactor with constant volume and pH control. Upon inoculation with fecal microbiota and a proper adaptation time of 2 weeks to ascending colon (AC) conditions, this reactor harbors a community that resemble that present in the AC [11,59]. Inoculum preparation, retention time, pH, and temperature settings were previously described [58]. The nutritional medium was composed as follows: arabinogalactan (1 g L^−1^), pectin (2 g L^−1^), xylan (1 g L^−1^), starch (5 g L^−1^), glucose (0.4 g L^−1^), yeast extract (3 g L^−1^), peptone (1 g L^−1^), mucin (4 g L^−1^), cysteine (0.5 g L^−1^). The fecal sample to start this SHIME experiment was derived from a healthy individual, who had no history of antibiotic treatment in the last year. The ethical approval to use human fecal samples to perform *in vitro* studies was granted by the Commission for Medical Ethics of UZ Gent (registration number B670201214538). After the reactor start up, the system was allowed to stabilize for 2 weeks before the start of the experiment [59]. The long-term experiment consisted of a 1-week control period in which the standard nutritional medium was administered to the model (condition A). After this, a treatment period of 1 week was performed in which the nutritional medium was supplemented with 4 g L^−1^ of yeast fermentate (condition B). To compensate for the additional administration of carbon sources, a corresponding amount of starch was removed. Two HMI modules, with a mucus layer of 250 μm, were connected to the AC vessel of the SHIME during the last three days of the control and of the treatment week. A constant flow of 6.5 mL min^−1^ (=3 dynes cm^−2^) of luminal suspension from/to the AC - by means of an 8-channel pump-head - (Figure 3) was maintained in the upper compartment. The medium in the lower compartment containing the enterocytes was replaced every 6 h by means of automatic pumping (8-channel pump-head), at a flow of 2 mL min^−1^. The exhausted medium was then collected from both the lower compartments of the HMI module to analyze the response of Caco-2 cells to the treatment in terms of production of inflammatory cytokines. For this purpose the samples were frozen at −20°C. The same samples collected at 6 (n = 4), 24 (n = 4) and 48 h (n = 2) were first used to measure the residual O_2_ concentration by means of a LDO probe. The HMI modules were maintained at a temperature of 37°C by means of a portable incubator (JP Selecta, Abrera, Spain).

To analyze the effect of the yeast fermentate on the microbial community composition, liquid samples were collected from the AC reactor during the control and treatment period (Figure 4). After 24 h and 48 h of incubation, a sterile blade was used to cut 6 cm^2^ of the membrane and mucus layer in the HMI module to collect samples to analyze the adhering bacteria. Samples were named as follows: A or B (control or treatment) + L or M (luminal or mucus compartment) + 0, 24 or 48 (time of incubation). Figure 4 shows a timeline of the experiment with relative sampling points.

### Biochemical and molecular analyses

*SCFA and ammonium production:* the microbial community activity in the AC was measured in terms of short-chain fatty acid (SCFA) and ammonium production as described by Van de Wiele et al. [60].

*Denaturing Gradient Gel Electrophoresis (DGGE):* the structure and composition of the microbial community was evaluated using DGGE on total bacteria, bifidobacteria and lactobacilli [60]. Metagenomic DNA was extracted from the L and M samples as previously described [61]. DGGE with a 45–60% denaturing gradient (50-65% for bifidobacteria) was used to separate the polymerase chain reaction (PCR) products obtained with a nested approach for the 16S rRNA genes of bifidobacteria (primers BIF164f-BIF662r) and lactobacilli (SGLAB0159f-SGLAB0667r). The first PCR round was followed by a second amplification with primers 338 F-GC and 518R. The latter primers were also used to amplify the 16S rRNA gene of all bacteria on total extracted DNA. The DGGE patterns obtained were subsequently analyzed using the Bionumerics software version 5.10 (Applied Maths, Sint-Martens-Latem, Belgium). In brief, the calculation of similarities was based on the Pearson (product–moment) correlation coefficient. Clustering analysis was performed using the unweighted pair group method with arithmetic mean clustering algorithm (UPGMA) to calculate the dendrograms of each DGGE gel. A cluster analysis was also performed on a composite dataset of all the gels with band-matching, Pearson correlation with standardized characters and bootstrap analysis with 1000 samplings.

*Quantitative PCR (qPCR):* Quantitative polymerase chain reaction (qPCR) for total bacteria, bifidobacteria, and lactobacilli were performed as reported by Possemiers et al. [62]. The qPCR for the Firmicutes and Bacteroidetes phyla was previously described by Guo et al. [63]; that for *Faecalibacterium prausnitzii* by Vermeiren et al. [64].

*Fluorescent in situ hybridization (FISH)*: 0.5 cm^2^ of the membrane were fixed in a solution containing 4% paraformaldehyde in phosphate buffered saline (pH7.2) and ethanol (1:1) at room temperature for 24 hrs and kept at 4°C for 5 days. The fixed membranes were subsequently embedded into paraffin wax blocks using standard laboratory techniques. Sections of 4 μm-thickness were cut off the paraffin blocks and were placed on StarFrost® slides (Waldemar Knittel Glasbearbeitungs- GmbH, Germany). To localize different groups of major intestinal bacterial, the obtained slides were hybridized with probes Bif164 for bifidobacteria and Fprau0645 for *Faecalibacterium prausnitzii* as described in Harmsen et al. [65]*.* To visualize all the bacteria, the hybridizations were combined with the universal Eub338 probe, labeled with either rhodamine or FITC to contrast the labels of the group-specific probes. These slides were visualized using a Leica Epi-fluorescence microscope (Leica, Germany) and a Zeiss, LSM 780 Confocal laser scanning microscopy (CLSM) (Zeiss Jena, Germany). The obtained pictures were evaluated using ImageJ software.

*Cytokines detection:* the supernatants from the cells compartments were assayed for the presence of interleukins IL-8 by using a commercially available ELISA kit and according to the manufacturer’s instruction (Quantikine ELISA, R&D Systems, Minneapolis, USA). Statistically significant differences of the treatment period, as compared to the average of the control period, were evaluated with a Student’s two-tailed t-test. Differences were considered significant if p ≤ 0.05.

## Competing interests

MM, BV, SP, PVdA, WV and TVdW are co-inventor of the pending patent WO2010118857A2.

## Authors’ contributions

MM, VB, SP, PVdA, WV and TVdW developed the concept of the HMI module and designed the experiments; MM performed all the microbiological experiments with the support of MSS and HH for the FISH analyses, of TH for the definition of the permeability of the module and of JP for the computational fluid dynamics simulation. BV and TDR performed all the work on the cell lines with the support of IP and with the help of LD for the cytokine analyses and MB for data interpretation and discussion. The manuscript was mainly handed by MM, BV and TVdW with a contribution from all the authors. All authors read and approved the final manuscript.

## Supplementary Material

Additional file 1: Figure S1 Computational fluid dynamics simulation of the module chamber under different shear forces. **Figure S2.** Clustering of DGGE fingerprinting analysis for total bacteria. Click here for file
