# Supplementary material for: The HMI™ module: a new tool to study the Host-Microbiota Interaction in the human gastrointestinal tract in vitro
Source: BMC Microbiol. 2014 May 22;14:133. doi: 10.1186/1471-2180-14-133 (PMC4039060; doi:10.1186/1471-2180-14-133)

The HMI™ module: a new tool to study the Host-Microbiota Interaction in the human gastrointestinal tract *in vitro*

Marzorati M.[1], Vanhoecke B.[2], De Ryck T.[2], Sadaghian Sadabad M.[3], Pinheiro I.[4], Possemiers S.[1,4], Van den Abbeele P.[1], Derycke L.[5], Bracke M.[2], Pieters J.[6], Hennebel T.[1], Harmsen H.J.M.[3], Verstraete W. [1], Van de Wiele T.[1*]

[1]*Laboratory of Microbial Ecology and Technology (LabMET), Ghent University, Coupure Links 653, B-9000 Gent, Belgium*

[2]*Laboratory of Experimental Cancer Research, Ghent University, 1P7, De Pintelaan 185, B-9000 Gent, Belgium*

[3]*Department of Medical Microbiology, University Medical Center Groningen, University of Groningen, Groningen, The Netherlands*

[4]*ProDigest, Technologiepark 3, 9052 Gent, Belgium*

[5]*Upper Airways Research Laboratory, Ghent University, Medical Research Building O, De Pintelaan 185, B-9000 Gent, Belgium*

[6]*Dept. of Biosystems Engineering, Ghent University, Coupure Links 653, B-9000 Gent, Belgium*

* For correspondence. E-mail: tom.vandewiele@UGent.be; Tel. +32 9 264 59 76; Fax +32 9 264 62 48.

Figure S1: computational fluid dynamics simulation of the module chamber under different shear forces. Color pathlines indicate the number of particle ID that will flow under different shear stresses. The experiment was designed to determine till which flow it was possible to obtain a homogenous flow. At low and medium shear stress no turbulence was observed and the flow was homogenous.

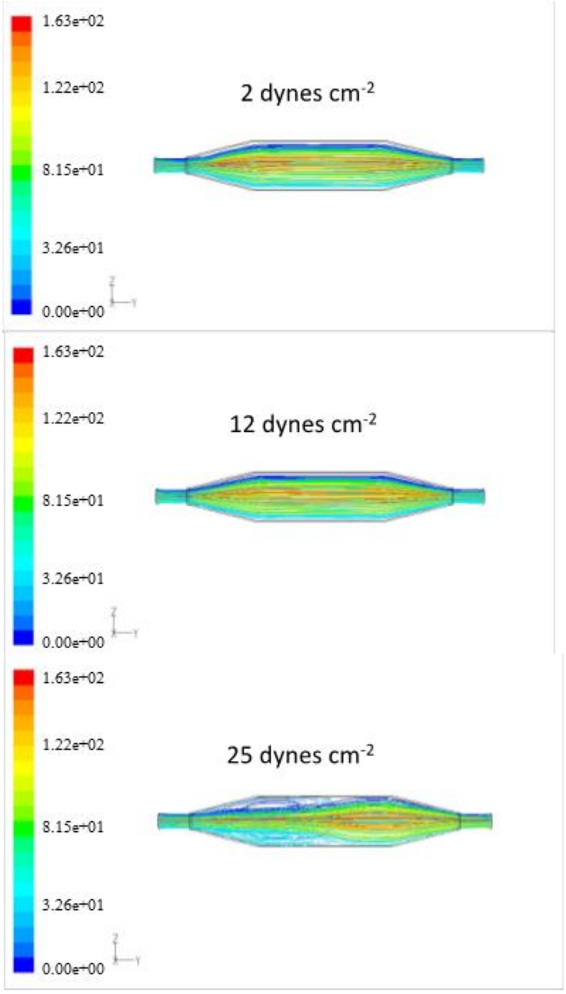

Figure S2: DGGE fingerprinting analysis for total bacteria. A = control period; B = treatment period. L = luminal samples; M = mucus sample. Clustering analysis was based on the Pearson product-moment correlation coefficient and the dendrogram was created by using UPGMA linkage.

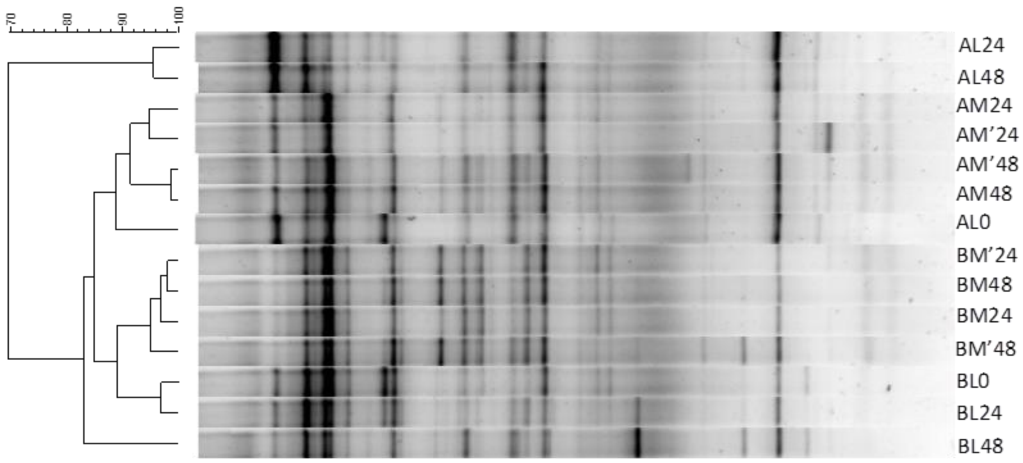

Supplement: Additional file 1: Figure S1 — Computational fluid dynamics simulation of the module chamber under different shear forces. Figure S2. Clustering of DGGE fingerprinting analysis for total bacteria. [file 1471-2180-14-133-S1.pdf]
